# Supplementary material for: RIP3 Translocation into Mitochondria Promotes Mitofilin Degradation to Increase Inflammation and Kidney Injury after Renal Ischemia–Reperfusion
Source: Cells. 2022 Jun 11;11(12):1894. doi: 10.3390/cells11121894 (PMC9220894; doi:10.3390/cells11121894)
Supplement: Supplementary file 1 [file cells-11-01894-s001.zip › cells-1724502-supplementary.pdf]

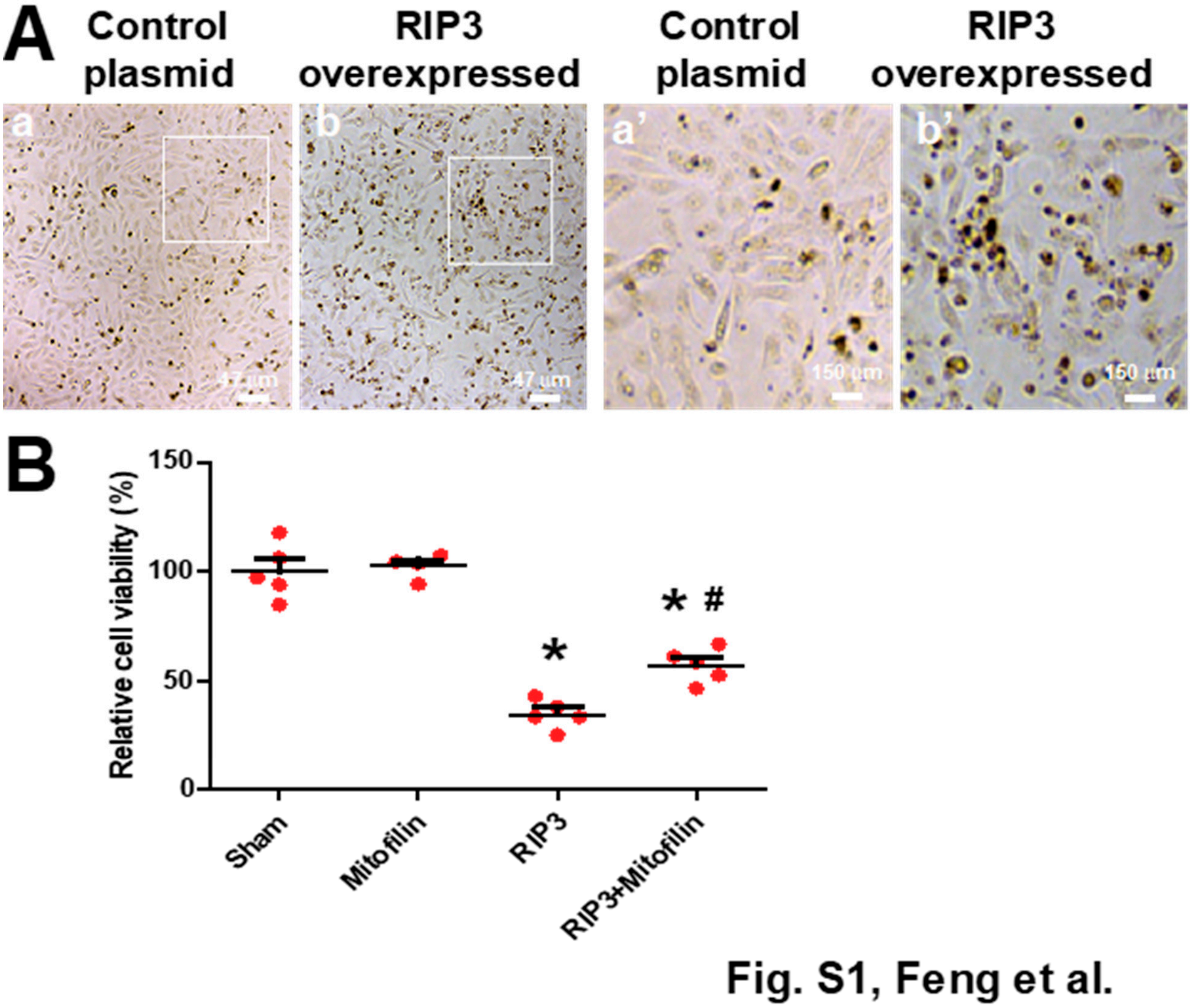

Figure S1. RIP3 overexpression in HK-2 cells increases cell death and reduces cell viability. (**A**) Image of HK-2 cells transfected with an RIP3-overexpressed plasmid and control vector (pCMV6), a′ and b′ are the regions magnified in a, and b, respectively. (**B**) Bar graph showing reductions in cell viability in RIP3-overexpressed, Mitofilin-overexpressed, and RIP3-overexpressed plus Mitofilin-overexpressed plasmids compared to the control (Ctrl) vector. The graph shows a significant reduction in cell viability in RIP3-overexpressed cells compared to the control plasmid. However, in cells co-transfected with both RIP3-overexpressed and Mitofilin-overexpressed plasmids, the level of cell viability was reduced when compared to cells transfected with RIP3-overexpressed vector alone. Note that there was no difference in the levels of cell viability in Mitofilin-overexpressed plasmid versus control plasmid. Values are expressed as means ±SEM; * P <0.05 versus control (Ctrl) vector group (n = 5/group).

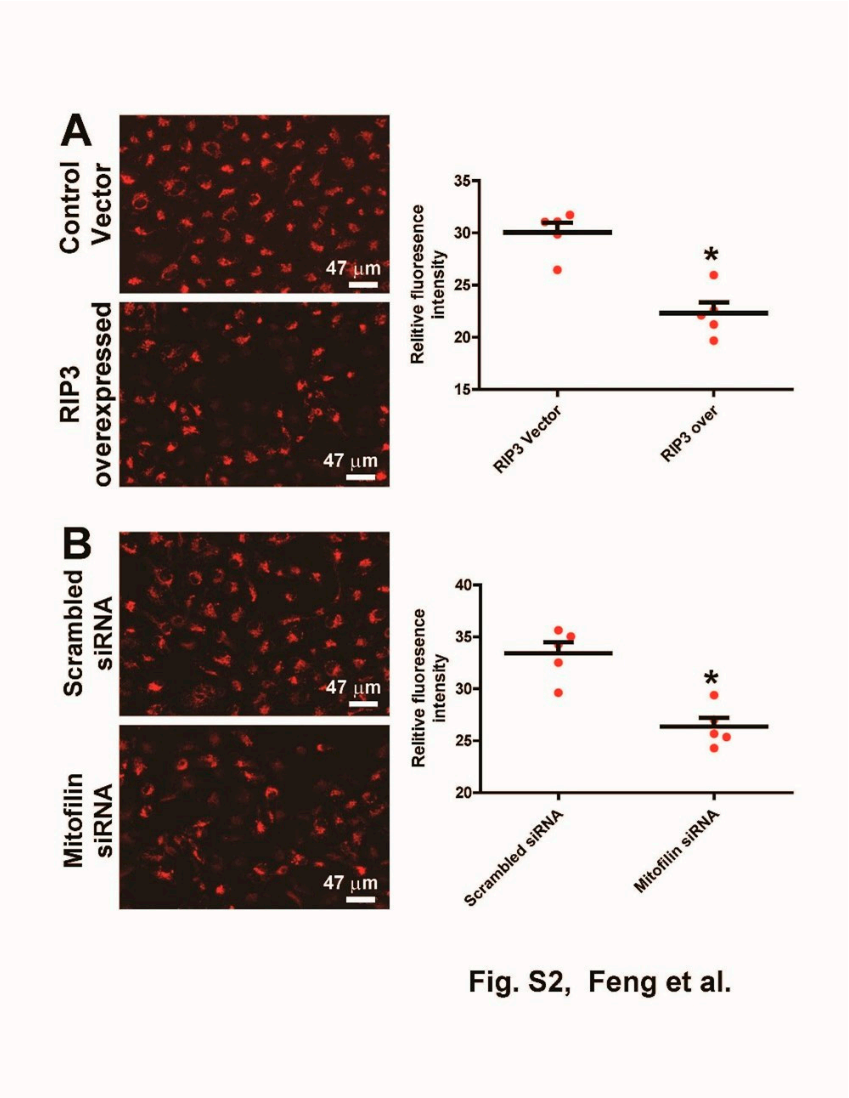

Figure S2. RIP3 overexpression or Mitofilin knockdown in HK-2 cells decreases mitochondrial membrane potential. (**A**) Left: Image of live HK-2 cells transfected with a control vector (pCMV6) or RIP3-overexpressed plasmid labeled with MitoTracker Red dye, respectively. Right: Bar graphs showing a reduction in mitochondrial membrane potential in cells transfected with RIP3-overexpressed plasmid compared to those transfected with control vector. Values are expressed as means ±SEM; * $P < 0.05$ versus control vector group (n = 5/group). (**B**) Left: Image of live HK-2 cells transfected with scrambled siRNA or with Mitofilin siRNA labeled with MitoTracker Red dye, respectively. Right: Bar graphs showing a reduction in mitochondrial membrane potential in cells transfected with Mitofilin siRNA compared to those transfected with scrambled siRNA. Values are expressed as means ±SEM; * $P < 0.05$ versus scrambled siRNA group (n = 5/group). Note that MitoTracker Red is a red fluorescent dye that stains mitochondria in live cells, and its accumulation is dependent upon membrane potential.
